# Gaps in recovery priorities between individuals with spinal cord injury and healthcare professionals

**DOI:** 10.1038/s44401-026-00073-4

**Published:** 2026-02-26

**Authors:** Soshi Samejima, Hajime Miyashita, Tatsuro Yamashita, Ryo Nakahara, Omer Faraz, Thomas Thordarson, Raza Malik, Rahul Sachdeva, Claire Shackleton, Andrei Krassioukov

**Affiliations:** 1 Department of Rehabilitation Medicine, University of Washington, Seattle, WA USA; 2 International Collaboration on Repair Discoveries (ICORD), University of British Columbia, Vancouver, BC Canada; 3 Department of Physical Therapy, Faculty of Rehabilitation, Morinomiya University of Medical Sciences, Osaka, Osaka, Japan; 4 Division of Rehabilitation, Sapporo Medical University Hospital, Sapporo, Hokkaido Japan; 5 Division of Physical Medicine and Rehabilitation, Faculty of Medicine, University of British Columbia, Vancouver, BC Canada; 6 Department of Physical Medicine & Rehabilitation, University of Kentucky, Lexington, KY USA; 7 Spinal Cord and Brain Injury Research Center (SCoBIRC), University of Kentucky, Lexington, KY USA; 8 University of Texas Health Houston, McGovern Medical School, Houston, TX USA; 9 Department of PM&R, UTHealth McGovern Medical School, Houston, TX USA; 10 GF Strong Rehabilitation Centre, Vancouver Coastal Health, Vancouver, BC Canada

**Keywords:** Health care, Neurology, Neuroscience

## Abstract

It is essential to align the perspectives of healthcare professionals on recovery priorities for the targeted population to promote health and well-being. This study aimed to identify the functional recovery priorities of individuals with spinal cord injury (SCI) and to assess gaps in recovery priorities between individuals with SCI and healthcare professionals. A web-based survey was conducted with 103 individuals with SCI and 85 healthcare professionals in Japan. Individuals with tetraplegia most frequently selected arm/hand function, while individuals with paraplegia more commonly selected bladder function. Comparing individuals with SCI and clinicians, individuals with SCI prioritized bladder and bowel function, whereas the healthcare professionals group assumed that individuals with SCI prioritized blood pressure control, mental health, and motor function. This study highlighted differences between individuals with SCI and clinicians, underscoring the need to include autonomic function in goal setting and to strengthen education for healthcare professionals in these areas.

## Introduction

Spinal cord injury (SCI) is a devastating condition resulting in a variety of physical disabilities and secondary complications, leading to decreased quality of life (QOL)^1,2^. The key study on priorities for recovery following SCI by Anderson reported that regaining arm/hand function is of high priority to persons with quadriplegia, whereas sexual function is of high priority to persons with paraplegia; however, bowel, bladder, and walking are of equal importance^3^.

Although the study was conducted nearly two decades ago, subsequent research has also reported that arm and hand function remained a high priority for individuals with tetraplegia^4–6^. However, among individuals with paraplegia, national differences have been observed, with walking ability in India^7^, pain and spasticity management in Korea^8^, and bladder and bowel function in China^9^ reported as the most prioritized recovery goals. Although the data from the Asian region are gradually accumulating, no comprehensive and quantitative study has yet investigated the recovery priorities of individuals with SCI in Japan.

Additionally, it is essential to understand the perspectives of healthcare professionals and caregivers on recovery priorities following SCI to inform healthcare. There is only one survey that reported several similarities and gaps between the perspectives of parents and caregivers, and children with SCI regarding priorities for their life and health domains after SCI^10^. In other conditions, healthcare professionals failed to accurately grasp patients’ priorities, resulting in a discrepancy between the two^11–13^. Such priority discrepancies can lead to care that is misaligned with the values of individuals with lived experience, resulting in reduced satisfaction. Similar challenges are likely to exist in SCI care and rehabilitation. To date, no study has simultaneously assessed and directly compared the recovery priorities reported by individuals with SCI with those that healthcare professionals assume from their perspectives.

Therefore, this survey study first aimed to identify the functional recovery priorities of individuals with SCI in Japan, and then examined how these priorities differ based on injury type—specifically, between individuals with tetraplegia and those with paraplegia. Furthermore, we assessed gaps in recovery priorities between individuals with SCI and healthcare professionals to promote healthcare and rehabilitation, aligning with patient values, and informing improvements in clinical education and practice.

## Results

### Participant inclusion and characteristics

A total of 219 responses were obtained from individuals with SCI, of which 115 were excluded due to missing, duplicate priority rankings, or lack of consent, resulting in 103 valid responses. In the HCP group, 173 responses were collected, with 88 excluded due to missing, duplicate priority rankings, or lack of consent, yielding 85 valid responses. The final analysis included 103 Japanese individuals with SCI (55 with tetraplegia, 48 with paraplegia) and 85 healthcare professionals.

Participant demographics and injury characteristics are summarized in Table 1. Among the SCI group, the neurological level of injury was distributed as follows: tetraplegia was observed in 55 individuals (53.4%), and paraplegia in 48 individuals (46.6%). The neurological level of injury was as follows: C1–C8, 55 individuals (53.4%), T1–T12, 36 individuals (35.0%), L1–L5, 12 individuals (11.7%), and S1–S4, none (0.0%), and no cases were unspecified (0.0%). The AIS grades were as follows: AIS A, 65 individuals (63.1%), AIS B, 8 individuals (7.8%), AIS C, 24 individuals (23.3%), AIS D, four individuals (3.9%), and AIS E, none (0.0%), and two cases unspecified (1.9%). Reported causes of injury included transport/motor vehicle accidents, 35 individuals (34.0%), sports/exercise-related incidents, 24 individuals (23.3%), diving accidents, 14 individuals (13.6%), falls, 3 individuals (2.9%), and other, 27 individuals (26.2%). In the HCP group, the professional composition was as follows: 47 physical therapists (55.3%), 20 occupational therapists (23.5%), 11 physicians (specialty specified) (12.9%), one nurse (1.2%), and five other professions (5.9%).

**Table 1:** Participant demographics and injury characteristics

Characteristics	Individuals with SCI (*n* = 103)	Healthcare professionals (*n* = 85)
Age, mean (SD), years	51.1 (13.8)	41.5 (9.9)
Sex, *n* (%)		
└Male/female	76 (73.8)/27 (26.2)	63 (74.1)/22 (25.9)
└Other/prefer not to answer	0 (0.0)	0 (0.0)
Injury-related		
Years since injury, mean (SD)	22.7 (15.1)	–
Paraplegia/tetraplegia, *n* (%)	48 (46.6)/55 (53.4)	–
Neurological level of Injury, *n* (%)		
└ C1–C8/T1–T12/L1–L5/S1–S4/unsure	55 (53.4)/36 (35.0)/12 (11.7)/0 (0.0)/0 (0)	–
AIS grade A/B/C/D/E/unsure, *n* (%)	65 (63.1)/8 (7.8)/24 (23.3)/4 (3.9)/0 (0.0)/2 (1.9)	–
Cause of SCI, *n* (%):		
└Motor vehicle accident/sports/diving/fall/other*	35 (34.0)/24 (23.3)/14 (13.6)/3 (2.9)/27 (26.2)	–
Profession, *n* (%)	–	
└Physician/nurse/PT/OT/Other/NA	–	11 (12.9)/1 (1.2)/47 (55.3)/20 (23.5)/5 (5.9)/1 (1.2)
└Acute or inpatient rehab/outpatient rehabilitation, or a community setting	–	74 (87.1)/11 (13.0)

*Other includes act of violence, degenerative condition, surgical complication, spinal stroke, spina bifida, tumor, birth defect, infection, arteriovenous malformation.

*SD* standard deviation, *SCI* spinal cord injury, *HCP* healthcare professional, *AIS* American Spinal Injury Association Impairment Scale; *PT* physical therapist, *OT* occupational therapist, *NA* not applicable.

### Functional priorities in individuals with SCI

**Fig. 1:**
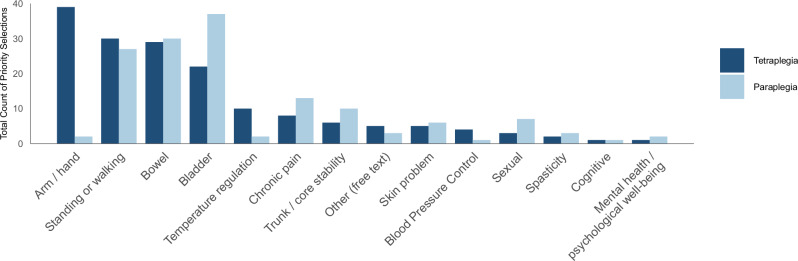
Ranking of priorities for functional recovery of individuals with tetraplegics and paraplegia. This bar graph compares the priorities for functional recovery based on the neurological level of individuals with spinal cord injury. Dark blue bars represent responses from participants with tetraplegia, and light blue bars represent responses from participants with paraplegia. Individuals with tetraplegia most prioritized arm and hand function, whereas those with paraplegia placed the highest priority on bladder function. Furthermore, a chi-square test revealed a statistically significant difference in the overall pattern of priority rankings between the two groups (*χ*² = 48.222, df = 13, *p* < 0.001).

A chi-square test compared priority rankings across 14 functional domains between individuals with tetraplegia and those with paraplegia. The analysis revealed a statistically significant difference between the two groups (*χ*² = 48.222, *df* = 13, *p* < 0.001, Fig. 1).

**Fig. 2:**
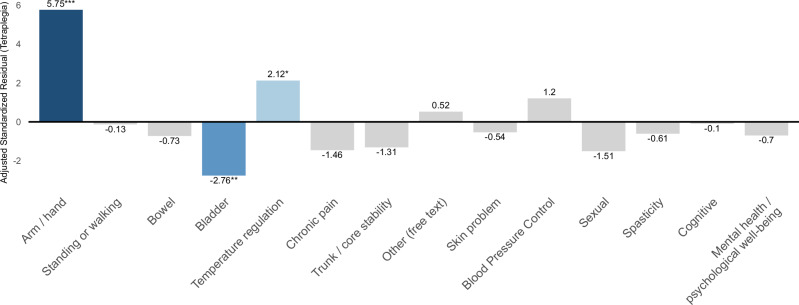
Adjusted standardized residuals for individuals with tetraplegia compared to individuals with paraplegia. This bar graph shows the deviation between the response tendencies of individuals with tetraplegia and the expected values, using adjusted standardized residuals. Significantly high residuals were observed for arm and hand function and temperature regulation. In contrast, negative residuals were found for functions such as bladder control. Statistical significance was determined based on adjusted standardized residuals exceeding ±1.96* (*p* < 0.05), ±2.58** (*p* < 0.01), or ±3.29*** (*p* < 0.001). Dark blue indicates large positive residuals. Blue indicates large negative residuals. Light blue represents small to moderate positive residuals, and light gray represents residuals close to zero.

Based on the results of the standardized residual analysis (Fig. 2), individuals with tetraplegia selected “Arm and hand function” significantly more frequently (AR = 5.75), while “Bladder function” was selected significantly less frequently (AR = −2.76). Additionally, “Temperature regulation” was also selected significantly more frequently (AR = 2.12) in individuals with tetraplegia. All of these differences were statistically significant at the 5% level. No other functional domains showed statistically significant differences between the groups.

### Gaps in priorities for functional recovery between individuals with spinal cord injury and healthcare professionals

**Fig. 3:**
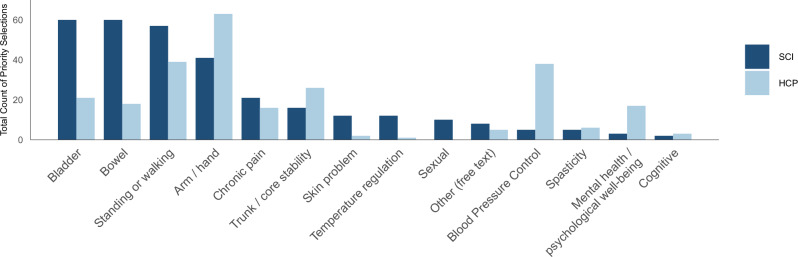
Ranking of priorities for functional recovery in individuals with spinal cord injury compared to healthcare professionals. This bar graph compares the priorities for functional recovery between individuals with spinal cord injury (SCI) and healthcare professionals (HCPs). Dark blue bars represent responses from participants with SCI, and light blue bars represent responses from HCPs. While individuals with SCI tended to prioritize the recovery of bladder, bowel, and sexual functions, HCPs were more likely to emphasize the importance of arm and hand function. Furthermore, a chi-square test revealed a statistically significant difference in the overall pattern of priority rankings between the SCI group and the HCP group (*χ*² = 122.86, df = 14, *p* < 0.001).

A chi-square test comparing functional recovery priorities between the SCI group, including individuals with both tetraplegia and paraplegia, and the HCP group (Fig. 3). The HCP group presented a significant group difference compared to the SCI group (*χ*² = 122.86, df = 14, *p* < 0.001).

**Fig. 4:**
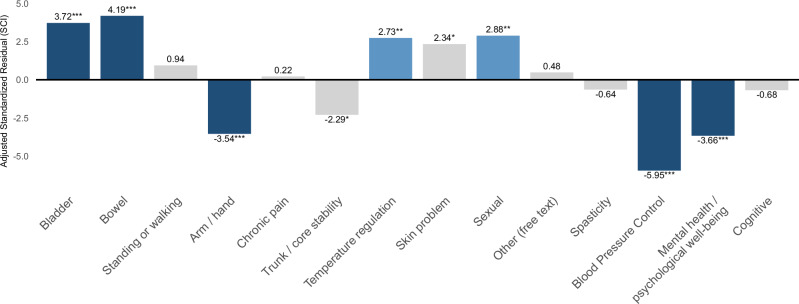
Adjusted standardized residuals for individuals with spinal cord injury compared to healthcare professionals. This bar graph illustrates the deviation between the response tendencies of individuals with SCI and the expected values, expressed as adjusted residuals. Significantly positive residuals were observed for the recovery of bladder, bowel, sexual function, and temperature regulation, whereas negative residuals were observed for blood pressure regulation, mental health, and arm/hand function. Statistical significance was determined based on adjusted standardized residuals exceeding ±1.96* (*p* < 0.05), ±2.58** (*p* < 0.01), or ±3.29*** (*p* < 0.001). Dark blue indicates large positive residuals. Blue indicates large negative residuals. Light blue represents small to moderate positive residuals, and light gray represents residuals close to zero.

Standardized residual analysis revealed that individuals with SCI selected “Bladder function” (AR = 3.72), “Bowel function” (AR = 4.19), “Temperature regulation” (AR = 2.73), “Skin problems” (AR = 2.34), and “Sexual function” (AR = 2.88) significantly more frequently than expected (Fig. 4). In contrast, the HCP groups selected “Arm/hand function” (AR = –3.54), “Trunk/core stability” (AR = –2.29), “Blood pressure regulation” (AR = −5.95), and “Mental health/psychological well-being” (A = –3.66) significantly more frequently compared to the SCI group. No significant group differences were observed for domains such as “standing or walking,” “chronic pain,” “spasticity,” or “cognitive function,” as all standardized residuals were within ±1.96.

## Discussion

This study revealed significant differences in recovery priorities depending on the neurological level of injury and the perspective of individuals with SCI and healthcare professionals (HCPs). Individuals with SCI tended to place greater emphasis on the recovery of autonomic functions such as bladder, bowel, sexual, and temperature regulation, while HCPs ranked blood pressure regulation, mental health, and arm and hand function as higher priorities. These findings highlight perceptual gaps between the lived experiences of individuals with SCI and the clinical perspectives of HCPs in the context of SCI care and rehabilitation.

Individuals with tetraplegia most frequently prioritized arm and hand function, whereas those with paraplegia most often selected bladder function. This trend is consistent with the pioneering study by Anderson et al., as well as reports from Asian countries such as South Korea, China, and India^3,7–9^. Additionally, bladder and bowel function, as well as standing and walking, were commonly selected as high-priority domains across both groups.

In individuals with tetraplegia, the restoration of arm and hand function is a highly critical determinant of independence in activities of daily living. Consequently, this domain tends to dominate their recovery priorities, which may lead to a relatively lower prioritization of other functions, including bladder function. In contrast, individuals with paraplegia retain upper-limb function, and therefore their most pressing daily challenges are less related to self-care and more closely associated with the burden and complexity of bladder management. As such, bladder function is more likely to emerge as a top priority in this group. This interpretation is consistent with previous research. Recovery of arm and hand function is the domain most strongly associated with improvements in QOL among individuals with tetraplegia, with 77% identifying it as the most important area for functional restoration^5^. Their findings indicate that, for individuals with tetraplegia, arm and hand function is prioritized above—or at least considered equally important as—bladder and bowel function.

However, it is noteworthy that in our study, bowel and bladder functions were ranked as high priorities following arm and hand function and standing and walking. This suggests that while the recovery of upper-limb function and mobility remains essential for independence, the burden of autonomic dysfunction—specifically bowel and bladder management—is recognized as a critical challenge of significant magnitude.

A growing body of evidence indicates that difficulties associated with bladder management constitute one of the major barriers to social participation among individuals with SCI. Recurrent urinary tract infections (UTIs), concerns about leakage, and the overall burden of managing neurogenic bladder have been shown to generate persistent anxiety and substantially restrict engagement in daily and community activities^14^. Similarly, the practical demands of bladder and bowel management frequently interfere with social, occupational, and interpersonal activities, leading individuals to limit their participation to avoid embarrassment or unpredictable situations^15^. Furthermore, quantitative studies have indicated that bladder dysfunction contributes to social isolation and diminished psychosocial well-being^16^.

Likewise, neurogenic bowel dysfunction (NBD) constitutes a substantial burden on QOL. Recent systematic reviews and meta-analyses have demonstrated that bowel dysfunction represents a significant lifestyle burden, negatively affecting multiple dimensions of daily living^17^. In particular, concerns related to fecal incontinence and the unpredictability of bowel movements have been identified as major stressors that limit social participation^18^. In addition, the time required for bowel care represents a practical barrier; a recent study reported that 74% of individuals with spinal cord injury spend >30 minutes per bowel care routine^19^. Taken together, the prolonged time commitment and psychological distress associated with bowel care substantially restrict social participation, mirroring the challenges observed with bladder management. Collectively, these findings suggest that, particularly among individuals with paraplegia who have achieved independence in upper-limb–related activities of daily living, the complexity, inconvenience, and health risks associated with bladder management—including recurrent UTIs—together with the time-consuming and psychologically burdensome nature of bowel management, may emerge as the most salient day-to-day concerns and represent the most significant barriers to social participation.

However, the priority for sexual function was at the 11th rank in individuals with tetraplegia and at the sixth rank in individuals with paraplegia, which was lower than the survey results in other studies. Japanese couples tend to be reluctant to talk about their sexual life^20,21^. This result is similar to the Korean study^8^. While this can be partially due to the difference in perceptions of sexual activity between Asian and Western cultures, further research needs to be performed to investigate the cultural impact on recovery priorities after SCI.

This study highlighted differences in functional recovery priorities between individuals with SCI and HCPs. Individuals with SCI tended to prioritize the recovery of bladder, bowel, and temperature regulation, whereas HCPs placed higher priority on blood pressure regulation, mental health, and arm and hand function. Bladder and bowel functions, which were frequently selected by individuals with SCI, are associated not only with potentially life-threatening complications such as UTIs and autonomic dysreflexia, but also with decreasing QOL^17,22^. These impairments represent persistent and intrusive challenges in daily life, creating barriers to employment, social relationships, and participation in community activities.

In contrast, the priorities for recovery collected from HCPs may be shaped by their clinical experience and medical knowledge, with a tendency to focus on managing complications such as orthostatic hypotension and autonomic dysreflexia that they encounter more frequently in clinical practice. These are not necessarily the functions most valued by individuals with SCI, but rather impairments that HCPs may perceive as having higher urgency and clinical significance^23,24^.

Furthermore, the results suggest that the injury profile envisioned by HCPs when responding was skewed. The high ranking of arm/hand function and blood pressure regulation implies that many HCPs may have envisioned individuals with tetraplegia rather than paraplegia. The high priority given to blood pressure regulation can also be partly explained by the fact that, among the 85 respondents of the HCP group, 67 respondents were rehabilitation professionals (47 PTs and 20 OTs), and 86.6% of these worked in acute care or inpatient rehabilitation settings. In these contexts, orthostatic hypotension is one of the most common complications encountered during rehabilitation and is well known to hinder progress^25^. Thus, the rankings of HCPs might be influenced by their experience in managing urgent symptoms rather than the expressed preferences of individuals with SCI. Overall, these findings indicate a potential mismatch between patient-centered goals and the perspectives of HCPs, offering important implications for goal setting and prioritization of interventions in SCI rehabilitation.

Several studies have compared the recovery priorities of patients with conditions other than SCI and those of HCPs^26,27^. They reported that patients with multimorbidity tended to focus on their present QOL, while clinicians emphasized the management of future risks. One systematic review comparing health outcomes and treatment priorities between patients with multimorbidity and clinicians demonstrated that patients most often prioritized “maintaining independence,” whereas clinicians prioritized extending life and risk reduction^27^. We observed clear differences in recovery priorities between Japanese individuals with SCI and HCPs, which can be improved for promoting healthcare and rehabilitation.

Previous studies on recovery priorities have compared individuals with SCI (e.g., tetraplegia vs. paraplegia), individuals with SCI and their parents/caregivers, patients with other health conditions and HCPs, and differences across HCP professions. However, no study has quantitatively compared the perspectives of individuals with SCI and HCPs simultaneously in Japan. This study could address this evidence gap. Future research should incorporate larger sample sizes and qualitative approaches to further elucidate how cultural background influences the formation of recovery priorities, thereby facilitating the implementation of healthcare and rehabilitation practices and educational curricula that truly reflect the values of individuals with lived experience.

This study has several limitations that should be acknowledged. First, study participants were primarily recruited through online platforms and professional networks using a convenience sampling approach, which may have introduced selection bias. Individuals who are more engaged with advocacy groups or academic communities, and who are more digitally literate, may have been overrepresented. Second, although important clinical variables such as time since injury, completeness of injury, and specific methods of bladder and bowel management were collected, subgroup analyses were not conducted due to substantial imbalances in sample sizes. This limitation, together with the relatively small sample size, restricted our ability to examine differences by AIS classification, time since injury, or HCP professional role, thereby limiting the generalizability of our findings. Future studies with larger and more balanced cohorts are warranted to explore these factors in greater detail. Third, a critical limitation is that the AIS classification was determined by participant self-report, which carries an inherent risk of misclassification. Because this study was conducted in a web-based survey format, a direct neurological examination by a clinician, which is the gold standard for AIS grading, was not feasible. This reliance on self-report may lead to inaccuracies, particularly as prior research indicates that self-reported AIS often aligns more closely with the initial injury grade than the current clinical status^28^. This limitation must be acknowledged when interpreting data related to injury severity. Fourth, although we applied objective quality-control criteria to exclude incomplete or invalid responses, the resulting sample showed differences in AIS distribution—specifically in AIS A and AIS C—between included and excluded participants. Therefore, the findings should be interpreted with caution, considering the possibility of sampling bias related to injury severity. Fifth, among the 85 HCP respondents, 67 respondents were rehabilitation professionals, and 58 of these (86.6%) worked in acute care or inpatient rehabilitation settings. As a result, the findings may disproportionately reflect the perspectives of rehabilitation specialists rather than the broader HCP community, and should be interpreted with this context in mind.

In conclusion, this study identified the perceptual gap between individuals with SCI and HCPs, providing direction for achieving rehabilitation centered on individuals with lived experience. In particular, it is essential to incorporate priorities for recovery related to autonomic functions—such as bladder, bowel, sexual function, and temperature regulation—into goal-setting processes. Enhancing educational curricula to address these domains will serve as a foundation for supporting both clinical and psychosocial recovery. Future multicenter, large-scale studies are warranted to further validate these findings and promote the implementation of rehabilitation practices grounded in the values of individuals with SCI.

## Methods

### Ethical considerations

This study was approved by the Clinical Research Ethics Board of the University of British Columbia (Approval Number: H21-01916) in accordance with the Declaration of Helsinki. Written informed consent was obtained from all participants prior to enrollment. We followed the American Association for Public Opinion Research (AAPOR) guidelines for survey method and quality^29^.

### Participants and recruitment

The study targeted two populations: individuals with spinal cord injury (SCI group) and healthcare professionals (HCP group). Inclusion criteria for the SCI group were as follows: (1) age ≥ 18 years, (2) Japanese nationality, and (3) the ability to provide informed consent and complete the questionnaire in Japanese. To confirm the diagnosis of SCI, the questionnaire included a screening item asking participants to report the cause of their injury (e.g., sports- or exercise-related accident, diving accident, fall, violence, traffic accident, fall from height, or postoperative complication). Participants were additionally asked to indicate their neurological status using the item: “Please indicate your AIS grade according to the American Spinal Injury Association Impairment Scale (AIS). Please refer to the explanations below.” A direct link to the AIS descriptions provided in the ISNCSCI worksheets was embedded to support accurate self-reporting. The AIS definitions were based on the 2019 revision of the International Standards for Neurological Classification of SCI (ISNCSCI), developed by the American Spinal Injury Association (ASIA) (Table 2)^30^, and were presented to participants as follows: AIS A (Complete): No sensory or motor function is preserved below the neurological level. AIS B (Sensory-Incomplete): Sensory but not motor function is preserved below the neurological level, including sacral segments. AIS C (Motor-Incomplete): Some motor function is preserved below the neurological level, but it is not functionally useful. AIS D (Motor-Incomplete): Motor function is preserved below the neurological level and is functionally useful. AIS E (Normal): Sensory and motor function below the neurological level is normal.

**Table 2:** American Spinal Injury Association Impairment Scale (AIS)

AIS	
A = Complete	No sensory or motor function is preserved in the sacral segments S4-5.
B = Sensory Incomplete	Sensory but not motor function is preserved below the neurological level and includes the sacral segments S4-5 (light touch or pin prick at S4-5 or deep anal pressure), AND no motor function is preserved more than three levels below the motor level on either side of the body.
C = Motor Incomplete	Motor function is preserved at the most caudal sacral segments for voluntary anal contraction (VAC), or the patient meets the criteria for sensory-incomplete status (sensory function preserved at the most caudal sacral segments S4-5 by LT, PP or DAP), and has some sparing of motor function more than three levels below the ipsilateral motor level on either side of the body. (This includes key or non-key muscle functions to determine motor-incomplete status.) For AIS C, less than half of the key muscle functions below the single NLI have a muscle grade ≥3.
D = Motor Incomplete	Motor incomplete status as defined above, with at least half (half or more) of key muscle functions below the single NLI having a muscle grade ≥ 3.
E = Normal	If sensation and motor function, as tested with the ISNCSCI, are graded as normal in all segments, and the patient had prior deficits, then the AIS grade is E. Someone without an initial SCI does not receive an AIS grade.

Using ND: to document the sensory, motor and NLI levels, the ASIA Impairment Scale grade, and/or the zone of partial preservation (ZPP) when they are unable to be determined based on the examination results.

Participants were excluded for the following reasons. First, individuals with a history of traumatic brain injury or cognitive impairment that could interfere with reliable questionnaire completion were excluded. Second, participants were excluded if key injury-related information was incomplete or invalid. This included missing values for Age at Time of Injury or Time Since Injury, as well as inappropriate entries in the Date of Injury field. Examples of inappropriate entries included a single digit such as “1,” descriptive statements such as “C5 spinal cord injury,” narrative comments such as “noticed symptoms around 2010” or “congenital,” and incomplete date formats such as “2006/2/,” “2010/10/,” or “2020/5/.” Finally, participants were excluded if priority-ranking items were missing or if duplicate rankings were identified. A duplicate ranking was defined as assigning multiple priority numbers to the same functional category or assigning the same priority number to multiple categories. For the HCP group, no restrictions were placed on professional discipline or years of experience. Eligible participants were defined as medical professionals who had at any point been involved in the clinical care of individuals with SCI. Recruitment was conducted through existing networks that researchers have in Japan. Study announcements were disseminated via institutional websites, social media platforms, and professional networks. In addition, collaborators and advocacy organizations were asked to assist with distribution.

### Questionnaire development

To assess participants’ priorities for functional recovery after SCI, a web-based survey was developed using the Qualtrics XM platform. Fourteen functional domains were selected based on prior literature^3,6,31^ and were refined through review by an international panel of SCI experts. Feedback from SCI advocacy organizations, such as Spinal Cord Injury BC and the International Spinal Research Trust, was incorporated to ensure patient-centered design. The initial questionnaire was written in English, then translated into Japanese using a forward–backward translation process by three bilingual clinician scientists to ensure linguistic accuracy and cultural relevance^32,33^. A pilot test was conducted with Japanese individuals with SCI to confirm clarity and usability prior to full-scale distribution.

### Questionnaire content

Participants first entered demographic and background information. For the SCI group, this included age, sex, cause of injury, neurological level, and American Spinal Injury AIS classification. For the HCP group, professional role and years of experience were recorded.

Participants were then asked to select and rank their top three priorities for functional recovery from the following 14 domains:

- Arm/hand function
- Bladder function
- Blood pressure control
- Bowel function
- Cognitive function
- Chronic pain
- Mental health/psychological well-being
- Sexual function
- Skin problems
- Spasticity
- Standing or walking
- Temperature regulation
- Trunk/core stability
- Other (free text)

To minimize response bias, the order of item presentation was randomized for each respondent. The survey was distributed online via secure URL and QR code access. All responses were automatically uploaded to a secure Qualtrics database hosted by the University of British Columbia. Data were anonymized and stored on password-protected computers accessible only to authorized research personnel. Data collection was conducted over approximately 11 months, from February 2024 to December 2024.

### Statistical analysis

Descriptive statistics, including means, standard deviations, frequencies, and percentages, were used to summarize participant demographics and injury characteristics. The SCI group was divided into two subgroups: individuals with tetraplegia and those with paraplegia. Frequencies of each selected functional domain were calculated within each subgroup.

To examine differences in functional recovery priorities between individuals with tetraplegia and paraplegia, as well as between individuals with SCI and HCPs, chi-squared tests of independence were conducted. In addition, adjusted standardized residual (AR) analysis was performed to identify functional domains that were selected significantly more or less frequently than expected within each group. Statistical significance was determined based on AR exceeding ± 1.96* (*p* < 0.05), ± 2.58** (*p* < 0.01), or ± 3.29*** (*p* < 0.001).

All statistical analyses were conducted using RStudio (Version 2025.05.0 + 496; The R Foundation for Statistical Computing)^34^.

### Ethical approval

The project was approved by the University of British Columbia (Approval number: H21-01916).

## Data Availability

The datasets analyzed during the current study are available from the corresponding author upon reasonable request.
